# Research on rapid imaging with cosmic ray muon scattering tomography

**DOI:** 10.1038/s41598-023-47023-w

**Published:** 2023-11-12

**Authors:** Qun-Gang Wen

**Affiliations:** Anhui University, Hefei, 23061 China

**Keywords:** Imaging techniques, Experimental particle physics

## Abstract

Cosmic ray muons tomography is a non-destructive imaging technique that uses the natural radiation of cosmic ray muons to create tomographic images of objects. This article presents a novel imaging algorithm that effectively utilizes experimental measurement data to achieve rapid and clear imaging of cosmic ray muons. A clear image can be obtained with only 20 min of measurement time and approximately 200 effective muons. However, the current detection flux is only about 0.044 cm$^{-2}$ min$^{-1}$, which is significantly lower than the natural cosmic ray flux of about 1 cm$^{-2}$ min$^{-1}$.

The universe is full of cosmic rays, including cosmic muons, which are high-energy particles that constantly bombard the Earth’s atmosphere. Muons are similar to electrons, but they are significantly heavier. This reduces their Bremsstrahlung and allows them to penetrate much deeper into matter. This unique property makes them an ideal probe for imaging the structures of dense materials. Muon scattering tomography (MST) is a technique that utilizes the scattering behavior of cosmic ray muons to estimate the material composition of a given volume, demonstrating significant potential for enhancing homeland security. The pioneering work on MST was conducted by the Los Alamos team in 2003^1^. Cosmic muons strike the sea level at a rate of about 10000 $\textrm{m}^{-2}\,\textrm{min}^{-1}$^2^. However, the detection efficiency of practical detection systems is far from this ideal value due to the multiple layers of detectors required for MST, as well as the limited detection area of the detectors and other factors. Therefore, the key to achieving fast imaging of cosmic muons lies in achieving imaging with low event counts.

Cosmis muons pass through the material, they are scattered by the coulomb potential of nuclei and eletrons. Each scattering will slightly affect the motion of the particles. The result of a large scattering process is that the charged particles will slightly deviate from the original direction of incidence. The scattering angle distribution of Coulomb scattering is described by Moliere theory^3^. For small-angle scattering, scattering angle is distributed near the mean value zero, which can be described by Gaussian distribution. The root-mean-square (RMS) width is related to the scattering material through its radiation length $L_0$ as follows:

1 $$\begin{aligned} \sigma _{\theta } = \frac{13.6\textrm{MeV}}{\beta pc}\sqrt{\frac{L}{L_0}}\Bigg [1+0.038 \ln \Bigg (\frac{L}{L_0}\Bigg )\Bigg ] \end{aligned}$$

where $\beta$ is the muon speed as a fraction of the speed of light, *pc* is the muon momentum in MeV/c, *L* is the path length of the muon through the material. The radiation length $L_0$ decreases rapidly as the atomic number of a material increases, and $\sigma _{\theta }$ increases accordingly. For a distribution with a mean value of zero, its RMS value can be obtained from the experiment data using the following equation.

2 $$\begin{aligned} \sigma ^2_{\theta } = \frac{1}{N}\sum ^{N}_{i=1}\theta ^{2}_{i} \end{aligned}$$

where the $\theta _{i}$ represents the measured scattering angle of the number *i* of muon events in the data, and *N* the total number of events. Large angle scattering from muons colliding with nuclei is similar to Rutherford scattering in that the distribution has a high tail (compared to Gaussian distribution). It is obvious that these large angle data have a great impact on the RMS value obtained in experiment. To reduce this impact of these large angle data, the so-called ratio algorithm was proposed in reference^4^. This algorithm focuses on small angle events, thereby improving the utilization efficiency of experimental data and effectively enhancing the final imaging quality. This paper further improves the efficiency of data utilization based on the algorithm, aiming to achieve high-quality imaging with fewer events.

To achieve experimental scattering-muon imaging, a set of muon detectors must be established above and below the object to be imaged. Each detector set includes two or more planes of position-sensitive muon detectors. The top set detects incoming muons while the bottom set detects outgoing muons. By analyzing the tracks of incoming and outgoing muons, one can obtain information on the scattering points and scattering angles of muons passing through the object. A tomographic reconstruction of the volume of interest is performed based on the data provided by multiple muons. The reconstruction area is divided into multiple voxels, and each voxel is assigned a value using a certain algorithm^6–8^. This method only uses the data passing through each voxel to calculate the corresponding value for that voxel. Therefore, in cases with small amounts of data, the size of the voxel needs to be relatively large to obtain sufficient statistical data. To maximize the utilization of experimental data, a ratio algorithm without voxel imaging has been proposed here, taking into account the characteristic of the ratio algorithm that focuses on small-angle data. In this method, each center position of the multiple voxels divided in the reconstruction region is called a reconstructed data point. Voxel-free means that the size of the voxel is infinitely large. Therefore, the ratio value calculation for each reconstructed data point will use all experimental data. The specific approach is as follows:

- Each reconstructed data point corresponds to a ratio value.
- To calculate the ratio value for a reconstructed data point, an attenuating function that is distance-dependent must be used to convert the experimental scattering angle to the reconstructed data point scattering angle based on the distance between the two points. For instance, if the scattering point of the experimental data point is located at position *a* with a scattering angle $\theta _{a}$, and the reconstructed data point is at position *A*, we obtain the angle for the reconstructed data point as $\theta _{Aa} = \theta _a\cdot \exp \bigg (-\frac{S^2_{Aa}}{2 k^2}\bigg )$, where $S_{Aa}$ is the distance between points *A* and *a*, *k* is a parameter calibrated based on actual data, and in this paper, we set *k* to be 1.6 cm.
- Using the ratio algorithm, we can obtain a corresponding ratio value for each reconstructed data point based on all of its reconstructed scattering angles. By visualizing all reconstructed data points and their corresponding ratio values within the measurement area, an image can be generated.

A prototype of muon tomography, called the muon Scattering and Transmission imaging facility ($\mu$STC)^5^, was developed. It included eight Micromegas detectors^9,10^, four front-end electronics cards (FECs), a general data acquisition (DAQ) board, a server, two scintillators for trigger generation, and several high-voltage (HV) modules. Each Micromegas detector achieved a spatial resolution better than 200 $\upmu$m and a detection efficiency higher than 90$\%$ for cosmic ray muons. With an active area of 15 cm $\times$ 15 cm, each detector recorded particle tracks at two positions in each of two orthogonal coordinates. The upper four Micromegas detectors recorded the tracks of incident muons, while the lower four Micromegas detectors captured the scattered tracks. When a muon passed through the active detector area, the FEC collected and sampled ionization signals. If a valid trigger signal was generated by the top and bottom scintillator planes, the signals were transmitted to the DAQ board. The distance between the two scintillator detectors was approximately 60 cm.

**Figure 1:**
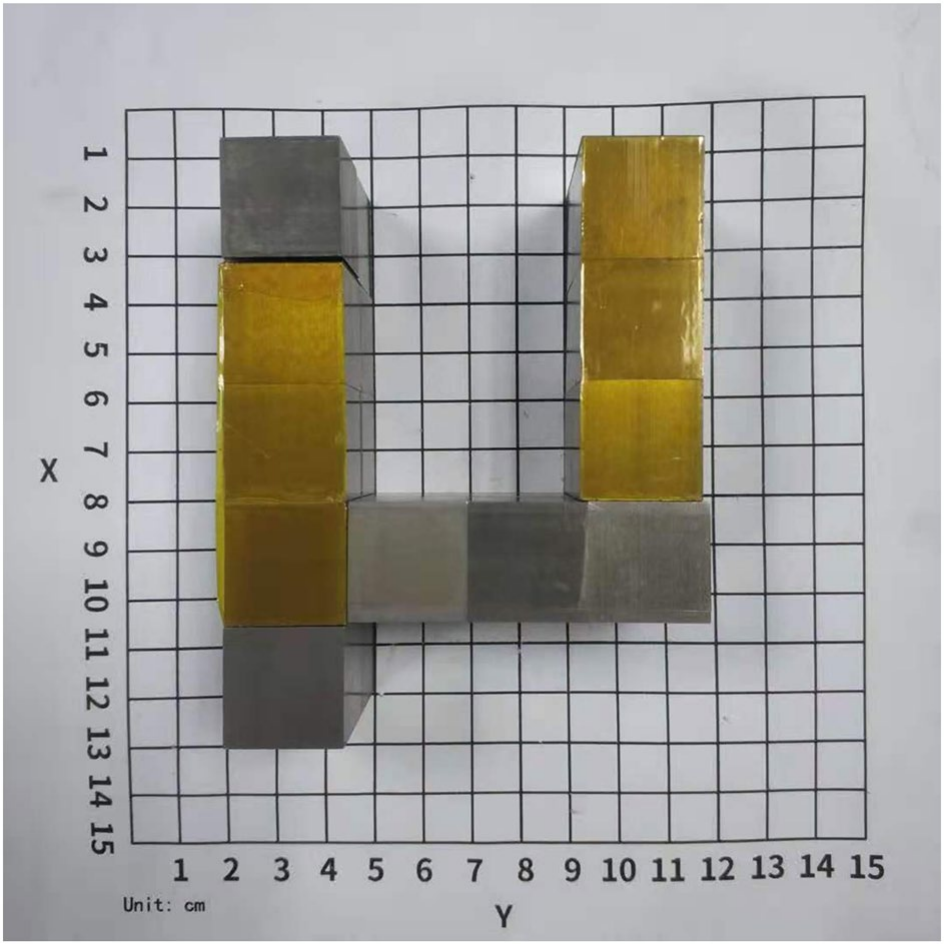
Photograph of a letter “$\mu$” sample under test.

A letter “$\mu$” was made of small tungsten cube as a test object, and the size of each cube was 2 cm $\times$ 2 cm $\times$ 2 cm (See Fig.1). In the vertical direction, the thickness of the sample was 4 cm. During online measurements, the system records measurement results in a file every 10 min. Each file typically contains around 100 effective muons that are utilized for the imaging algorithm. The imaging results obtained using the ratio algorithm without voxel imaging are shown in Fig. 2. To highlight the high Z material portion, a colormap is used in the figure to map the values of $S_i$ , which are inversely related to the ratio values. The calculation is as follows:

3 $$\begin{aligned} S_i = \frac{\frac{1}{R_i} - \Big \langle \frac{1}{R}\Big \rangle }{max(\frac{1}{R}) - min(\frac{1}{R})} \end{aligned}$$

Here, $R_i$ represents the ratio value corresponding to reconstruction point *i*, $\Big < \frac{1}{R}\Big>$ represents the average of the inverse ratio values, $max \left( \frac{1}{R}\right)$ refers to the maximum inverse ratio value, and $min \left( \frac{1}{R}\right)$ represents the minimum inverse ratio value. In data visualization, the colormap range is restricted to [0, 0.5$max \left( \frac{1}{R}\right)$]. Additionally, the size of the reconstruction points is proportional to $S_i$. From top to bottom, the images correspond to the imaging results after 60 min, 30 min, and 20 min of measurements, respectively. The figure demonstrates that a relatively clear image of the letter $\mu$ can be obtained with just 20 minutes of measurement time and around 200 effective muons. The current detection system has a detection flux of about 0.044 cm$^{-2}$ min$^{-1}$, which is significantly lower than the natural cosmic ray muon flux of about 1 cm$^{-2}$ min$^{-1}$. However, there is a significant potential for enhancing the detection flux of the system in the future. By increasing the detection flux, muon imaging can be achieved in shorter time periods. This would be particularly beneficial for practical applications in homeland security, specifically in detecting special nuclear materials such as contraband Uranium in cargos and containers.

**Figure 2:**
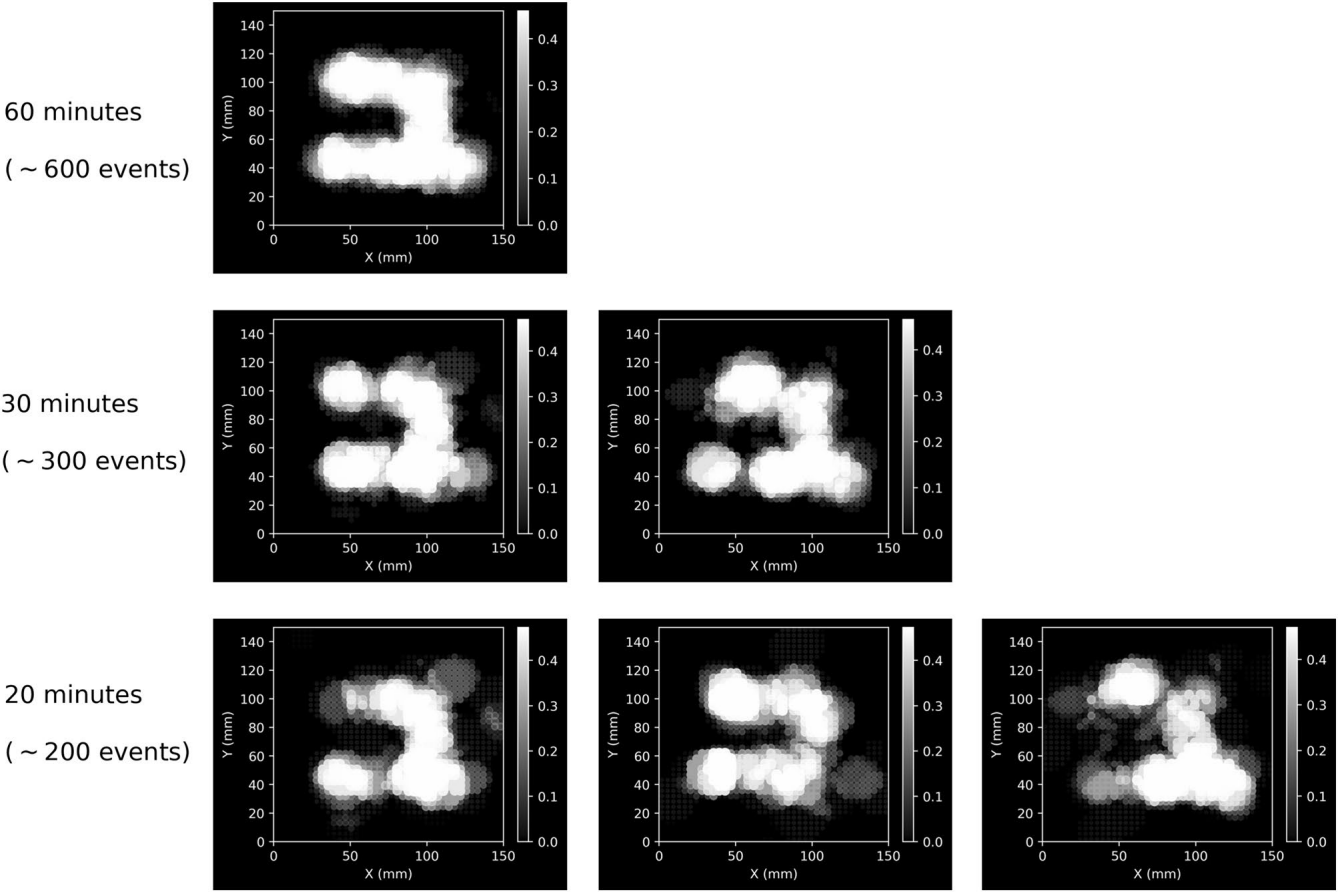
Tomography results after 60 min, 30 min and 20 min.

### Supplementary Information

Supplementary Information.

## Data Availability

The datasets used and analysed during the current study available from the corresponding author on reasonable request.
