# Supplementary material for: Research on rapid imaging with cosmic ray muon scattering tomography
Source: Sci Rep. 2023 Nov 12;13:19718. doi: 10.1038/s41598-023-47023-w (PMC10641068; doi:10.1038/s41598-023-47023-w)
Supplement: Supplementary file 1 — Supplementary Information. [file 41598_2023_47023_MOESM1_ESM.zip › supplementary_files/ReadMe.pdf]

Simple application of ratio algorithm for voxel-free imaging

Wen Qun-Gang (Email: qungang@ahu.edu.cn)

The algorithm introduced in this article differs somewhat from previous ones, so it is recommended to use a practical application to aid understanding. Here, we provide a simple application example of the ratio algorithm for voxel-free imaging, which includes three files: poca.txt, Simple_Apply.cxx, and show_image.py. poca.txt is a set of real experimental measurement data from a 20-minute experiment, with 219 measurement events. The first three columns of the list correspond to the x, y, and z coordinates of the scattering points (unit: millimeters), and the fourth column is the angle value between the muon incident and exit rays (unit: degrees). show_image.py is a Python program for displaying the imaging results. In a Linux system, you can use the command line to display the scatterplot imaging of poca.txt by running the following command:

```
python show_image.py poca.txt
```

Simple_Apply.cxx is a C++ program that uses the ratio algorithm for voxel-free imaging to generate a new imaging file called Use_Data_rebuilt.txt from the data in poca.txt. In this example, the detection region is a cuboid of 150mm $\times$ 150mm $\times$ 160mm, with the lowest point in the z direction being 160mm. The "radius" in the non-volume imaging is an adjustable parameter, as is the "theta_cut" in the ratio algorithm, which can be adjusted according to the actual situation. The reconstructed points in the file Use_Data_rebuilt.txt are obtained by taking 60,000 random points within the detection region. The number of reconstructed points can be adjusted by modifying the value of "re_num". To use this program in a Linux system, you can follow these steps:

1. In a terminal, enter the following command:

   ```
   g++ Simple_Apply.cxx -o Simple_Apply
   ```

   This will generate an executable file named Simple_Apply.

2. In the same directory as poca.txt, run the following command:

   ```
   ./Simple_Apply
   ```

   This will generate the Use_Data_rebuilt.txt file. The first three columns of the file correspond to the x, y, and z coordinates of the reconstructed data points, with units of millimeters. The fourth column contains the reciprocal of the ratio value calculated using the ratio algorithm for voxel-free imaging for each reconstructed point.

3. Finally, in the terminal, enter the following command:

   ```
   python show_image.py Use_Data_rebuilt.txt
   ```

   This will display the imaging results using the data in the Use_Data_rebuilt.txt file.
